# MixTwice: large-scale hypothesis testing for peptide arrays by variance mixing

**DOI:** 10.1093/bioinformatics/btab162

**Published:** 2021-03-08

**Authors:** Zihao Zheng, Aisha M. Mergaert, Irene M. Ong, Miriam A. Shelef, Michael A. Newton

**Affiliations:** Department of Statistics, University of Wisconsin-Madison, Madison, WI 53706, USA; Department of Medicine, University of Wisconsin-Madison, Madison, WI 53705, USA; Department of Medicine, University of Wisconsin-Madison, Madison, WI 53705, USA; Department of Pathology and Laboratory Medicine, University of Wisconsin-Madison, Madison, WI 53705-2281, USA; Department of Biostatistics and Medical Informatics, University of Wisconsin-Madison, Madison, WI 53726, USA; Department of Obstetrics and Gynecology, University of Wisconsin-Madison, Madison, WI 53792, USA; University of Wisconsin Carbone Comprehensive Cancer Center, University of Wisconsin-Madison, Madison, WI 53792, USA; Department of Medicine, University of Wisconsin-Madison, Madison, WI 53705, USA; William S. Middleton Memorial Veterans Hospital, Madison, WI 53705, USA; Department of Statistics, University of Wisconsin-Madison, Madison, WI 53706, USA; Department of Biostatistics and Medical Informatics, University of Wisconsin-Madison, Madison, WI 53726, USA; University of Wisconsin Carbone Comprehensive Cancer Center, University of Wisconsin-Madison, Madison, WI 53792, USA

## Abstract

**Summary:**

Peptide microarrays have emerged as a powerful technology in immunoproteomics as they provide a tool to measure the abundance of different antibodies in patient serum samples. The high dimensionality and small sample size of many experiments challenge conventional statistical approaches, including those aiming to control the false discovery rate (FDR). Motivated by limitations in reproducibility and power of current methods, we advance an empirical Bayesian tool that computes local FDR statistics and local false sign rate statistics when provided with data on estimated effects and estimated standard errors from all the measured peptides. As the name suggests, the MixTwice tool involves the estimation of two mixing distributions, one on underlying effects and one on underlying variance parameters. Constrained optimization techniques provide for model fitting of mixing distributions under weak shape constraints (unimodality of the effect distribution). Numerical experiments show that MixTwice can accurately estimate generative parameters and powerfully identify non-null peptides. In a peptide array study of rheumatoid arthritis, MixTwice recovers meaningful peptide markers in one case where the signal is weak, and has strong reproducibility properties in one case where the signal is strong.

**Availabilityand implementation:**

MixTwice is available as an R software package https://cran.r-project.org/web/packages/MixTwice/.

**Supplementary information:**

Supplementary data are available at *Bioinformatics* online.

## 1 Introduction

Peptide microarray technology is used in biology, medicine and pharmacology to measure various forms of protein interaction. Like other microarrays, a peptide array contains a large number of very small probes arranged on a glass or plastic chip. Each probe occupies a spatial position on the array and is comprised of many molecular copies of a short amino-acid sequence (a peptide) anchored to the surface, perhaps 12–16 amino acids in length, depending on the design. In antibody profiling experiments, the array is exposed to serum derived from a donor’s blood sample; antibodies in the sample that recognize an anchored peptide epitope may bind to the probe. In order to measure these antibody/antigen binding events, a second, fluorescently tagged antibody is applied, which binds to exposed sites on the already-bound antibodies, providing quantitative readout at probes where there has been sufficient binding of serum antibody recognizing the peptide epitopes. High-density peptide microarrays have emerged as a powerful technology in immunoproteomics, as they enable simultaneous antibody binding measurements against millions of peptide epitopes. Such arrays have guided the discovery of markers for viral, bacterial and parasitic infections (Bailey *et al.*, 2020; Mishra *et al.*, 2018; Tokarz et al., 2020) and have illuminated the serological response to cancer (Yan et al., 2019) and cancer immunotherapy (Hoefges *et al.*, 2020). The photolithographic design allows for custom arrays, which have benefited studies of autoimmunity, for example, where various forms of post-translational modification (e.g. citrullination) create targets for autoantibodies (Bailey *et al.*, 2017; Zheng et al., 2020).

The high dimensionality and small sample size of many peptide array experiments challenge conventional statistical approaches. Zheng *et al.* (2020), for example, reported a custom peptide array having 172 828 distinct features and array data from 60 human subjects across several disease subsets. This dimensionality is relatively high compared to gene expression studies, but quite low compared to other peptide array studies; arrays that probe the entire human proteome carry over 6 million peptide features, for example. Methods for large-scale hypothesis testing respond to these challenges, often aiming to control the false discovery rate (FDR) (e.g. Efron, 2012). FDR-controlling procedures are more forgiving than techniques that control the probability of any type I errors (e.g. Bonferroni correction), but they still extract a high penalty for dimensionality in the peptide array regime involving 10^5^–10^6^ features. When additional data are available, it may be possible to further limit penalties associated with large-scale testing.

Continuing with Zheng *et al.* (2020), the authors sought to identify peptides for which antibody binding levels differ between control subjects and rheumatoid arthritis (RA) patients expressing a specific disease marker combination [cyclic citrullinated peptide (CCP)^+^ and rheumatoid factor (RF−)]. Sera from 12 subjects in each group were applied to their custom-built array. After pre-processing, a univariate statistic (*t*-statistic) measured statistical changes at each peptide. Peptides with the most extreme statistics (and smallest *P*-values) would be set aside for further validation. In the CCP^+^ RF− RA example, no peptides had a FDR-adjusted *P*-value <10% by either the Benjamini–Hochberg (BH) method (Benjamini and Hochberg, 1995) or the more sensitive *q*-value method (Storey, 2003), although the latter method estimated that 21% of the peptides in fact have differential binding between the two groups.

Improving power while maintaining robustness and reproducibility is a theme of contemporary large-scale inference that we explore in the peptide array setting. The BH and *q*-value procedures yield no discoveries in the CCP^+^ RF− RA example at one conventional FDR level. If this is due to low statistical power, it may not be surprising since these procedures enter quite late in data analysis, after all *P*-values have been computed. Procedures that intervene earlier have access to more information, and thereby may have better overall operating characteristics. Efron’s local FDR approach, locFDR, intervenes on test statistics just prior to *P*-value computation and has improved power properties in some settings (Efron *et al.*, 2001). Independent filtering combines a selection statistic, such as marginal sample variance, and then applies an FDR-controlling procedure to the selected peptides (Bourgon *et al.*, 2010). Neither locFDR nor independent filtering at 50% yielded any results in the CCP^+^ RF− RA example, as it happens. We have the same null finding by independent hypothesis weighting (IHW), which generalizes independent filtering in not requiring a specific selection rate (Ignatiadis *et al.*, 2016).

Adaptive Shrinkage (ASH) is a recent innovation for large-scale testing that intervenes after each peptide yields both an estimated effect and an estimated standard error (Stephens, 2017). There are several variations of its empirical Bayesian formulation; when using the *t*-distribution sampling model version of ASH (say ASH-t), we discover 76 peptides to have differential antibody binding in the CCP^+^ RF− RA comparison, also at 10% FDR control. This may reflect increased power and is consistent with numerical studies showing increased power of ASH in many settings. A recent report from Professor Stephens’s group points out a technical limitation of ASH-t that could cause FDR inflation. It proposes a two-step ASH procedure that pre-processes the standard error estimates and then follows with the ASH-t procedure on modified input (Lu and Stephens, 2019). It happens that we discover 12 peptides with differential binding affinity by two-step ASH at 10% FDR. The different behavior of FDR-controlling procedures in the CCP^+^ RF− RA example exposes ongoing practical challenges that are also revealed in comprehensive numerical studies (Korthauer *et al.*, 2019).

Data analysts face many issues as they filter high-dimensional measurements into short lists for experimental follow-up. In studying this problem, we propose and evaluate a flexible empirical Bayesian mixture method that, like ASH, intervenes after effect estimates and standard errors are computed on each testing unit. The proposed MixTwice procedure involves shape-constrained mixture distribution for latent effects and also a separate non-parametric mixture for variance parameters (Section 2). We leverage existing tools for constrained optimization in order to estimate the underlying mixing distributions, and we present a variety of comparative numerical experiments on the operating characteristics of MixTwice. The CCP^+^ RF− RA peptide array example happens to yield 44 peptides having significant differential antibody binding at 10% FDR. A closer look at the identified peptides reveals binding patterns consistent with other biological information about RA, and thus provides a measure of confidence that these discoveries are not artifacts. In a second RA example where differential signals are stronger, MixTwice shows a higher level of reproducibility than other approaches when presented with two independent datasets on the same populations.

## 2 Mixture model

We index peptides by i=1,2,…,m and suppose that the two-group peptide array data have been obtained and pre-processed in order to yield two summary statistics per peptide: (*x_i_*, *s_i_*). The first component, *x_i_*, is an estimated effect. It measures the difference between the two groups, such as a difference in sample means of log-transformed data, and is viewed as statistical estimate of an underlying effect, say *θ_i_*. In this view, *x_i_* is a random variable having some sampling distribution, which we take to be Gaussian centered at *θ_i_*; this is warranted noting the behavior of suitably transformed fluorescence measurements coupled with central-limit effects for modest to large sample sizes. The second component, *s_i_*, is an estimated standard error. In the Gaussian sampling model, $E(x_{i})=\theta_{i}$ and var$(x_{i})=\sigma_{i}^{2}$, and $s_{i}^{2}$ is a sample-based estimate of the variance $\sigma_{i}^{2}$. We seek inference about the value of *θ_i_* using local data (*x_i_*, *s_i_*) as well as data $\left\{(x_{i\prime },s_{i\prime })\right\}$ from all peptides, which informs the distribution of effect and variance parameters across the array.

Our formulation is common in large-scale inference, and we could infer *θ_i_* values in a number of ways. For example, we could produce a peptide-specific *P*-value from the test statistic $t_{i}=x_{i}/s_{i}$ against the null hypothesis $H_{0,i}:\theta_{i}=0$. We might refer *t_i_* to a Student-*t* distribution, obtain a two-sided *P*-value, and then process the *P*-values through the BH or *q*-value methods to adjust for multiplicity (Benjamini and Hochberg, 1995; Storey, 2003). Alternatively, we might use the collection $\left\{t_{i}\right\}$ and model their fluctuations as a discrete mixture of null and non-null cases, as in the locFDR procedure (Efron *et al.*, 2001; Strimmer, 2008). Both locFDR and *q*-value methods are based upon discrete mixtures; interestingly, the reduction of *t_i’_*s to two-sided *P*-values entails a loss of sign information that is enough to reduce statistical power in some settings. A more ambitious approach goes beyond null/non-null mixing to allow a full probability distribution of effects *θ_i_* in order to account for fluctuations across all the peptides. ASH is appealing because it acquires robustness through a non-parametric treatment of this distribution, say g(θ), while using reasonable shape constraints to regularize the estimation (Stephens, 2017). Power advantages of ASH over other methods stem in part from its use of more data per peptide.

In the context of an estimated mixture model there are two useful empirical-Bayesian inference statistics. The first is local FDR (lfdr), $l_{i}=P(\theta_{i}=0|x_{i},s_{i}^{2})$. The term *local* FDR was coined by Professor Efron, and the statistic may be computed in various settings beyond the specific mixture deployed in Efron *et al.* (2001). The list ℓ of statistically significant peptides will be $\ell =\{i:l_{i}\leq c\}$ for some threshold *c*. Notably, small *l_i_* warrants peptide *i* to be placed in ℓ; but the value *l_i_* is also the probability (conditional on data) that such placement is erroneous (Newton *et al.*, 2006). Given the data, the expected rate of false discoveries in ℓ is dominated by *c*. The local false sign rate (lfsr) is analogous to lfdr, but it avoids relying on effects being precisely zero; when the estimated effect is positive, for example, the lfsr is $P(\theta_{i}\leq 0|x_{i},s_{i}^{2})$. Lists controlling lfsr may be constructed in the same way as ℓ, and may be slightly smaller for the same value of *c*. (In the CCP^+^ RF− RA example in Section 1, ASH lfsr and lfdr lists are the same at the 10% level.)

With modest sample sizes, differences between estimated standard errors $\left\{s_{i}\right\}$ and actual standard errors $\left\{\sigma_{i}\right\}$ can affect the performance of existing tools for lfdr and lfds. To better account for these differences, we propose an additional mixture layer involving a sampling model $p(s_{i}^{2}|\sigma_{i}^{2})$, which we derive from normal-theory considerations, and a flexible nonparametric mixing distribution $h(\sigma^{2})$. For both nonparametric components—*g* on effects *θ_i_* and *h* on squared standard errors $\sigma_{i}^{2}$—we use finite grids and treat each distribution as a vector of probabilities. We estimate *g* and *h* by maximum likelihood, respecting unimodal shape constraints for *g* (as in ASH), but otherwise allowing any distributional forms.

Suppose that effects take values in a finite, regular grid $\left\{a_{-K},a_{-K+1},\ldots ,a_{0},a_{1},\ldots ,a_{K}\right\}$ where *a*_0_ is the presumed mode, taken to be $a_{0}=0$ in typical applications in which we aim to retain the null hypothesis of no group difference. We use *K *=* *15 in numerical work reported here. Unimodality of the mixing distribution $g=(g_{k})$ is expressed as a set of ordering constraints: $g_{k}\geq g_{k+1}$ for k=0,1,…,K and $g_{k}\leq g_{k+1}$ for k=−K,−K+1,…,−1. We also set a second regular grid $\left\{0<b_{1},b_{2},\ldots ,b_{L}\right\}$ for squared standard errors, and impose no constraints on the mixing distribution $h=(h_{l})$ aside from the basic nonparametric essentials: $h_{l}\geq 0$ and $\sum_{l}h_{l}=1$.

The contribution to the likelihood objective from peptide *i* is $p(x_{i},s_{i}^{2}|g,h)$:

(1) $$\begin{array}{c} =\sum_{k}\sum_{l}P(\theta_{i}=a_{k})P(\sigma_{i}^{2}=b_{l})p(x_{i},s_{i}^{2}|\theta_{i}=a_{k},\sigma_{i}^{2}=b_{l})\\ =\sum_{k}\sum_{l}g_{k}h_{l}p(x_{i}|\theta_{i}=a_{k},\sigma_{i}^{2}=b_{l})p(s_{i}^{2}|\sigma_{i}^{2}=b_{l})\\ =\sum_{k}\sum_{l}g_{k}h_{l}\frac{1}{\sqrt{b_{l}}}\phi \left(\frac{x_{i}-a_{k}}{\sqrt{b_{l}}}\right)\frac{\nu }{b_{l}}\chi_{2,\nu }\left(\frac{\nu s_{i}^{2}}{b_{l}}\right) \end{array}$$

where ϕ is the standard normal probability density, $\chi_{2,\nu }$ is the density of a chi-square random variable on *ν* degrees of freedom. Under a normal data model, *ν* is determined by design (e.g. total samples minus two in the traditional two-sample comparison). The chi-square model is accurate asymptotically for a wide range of non-normal sampling distributions, however the degrees of freedom needs estimation in these cases (O’Neill, 2014).

To estimate the mixing distributions *h* and *g*, we use the log-likelihood objective function, with terms as in (1). In MixTwice, we solve the constrained optimization:

(2) $$\begin{array}{c} \underset{g,h}{\min}-l(g,h)=-\sum_{i=1}^{m}\;\text{log}\;p(x_{i},s_{i}^{2}|g,h)\\ \text{Subject}\;\text{to:}\;\;g_{k},h_{l}\geq 0\;\forall k,l\\ \sum_{k}g_{k}=\sum_{l}h_{l}=1\\ g_{k}\leq g_{k+1},\;k\in \{-K,-K+1,\ldots ,-1\}\\ g_{k}\geq g_{k+1},\;k\in \{0,1,\ldots ,K\} \end{array}$$

The gradient and Hessian of *l*(*g*, *h*) are readily available, and so (2) may be solved efficiently using augmented Lagrangian for constrained optimization, using the Broyden–Fletcher–Goldfarb–Shanno algorithm (BFGS) algorithm for inner loop optimization, which is implemented in the R package alabama (Varadhan, 2015). We extract lfdr and lfsr statistics from the peptide-specific posterior distributions at the optimized vectors $\hat{g},\hat{h}$: $P(\theta_{i}=a_{k}|x_{i},s_{i}^{2})$ 
 

(3) $$\begin{array}{c} =\sum_{l}P(\theta_{i}=a_{k},\sigma_{i}^{2}=b_{l}|x_{i},s_{i}^{2})\\ \propto {\hat{g}}_{k}\sum_{l}{\hat{h}}_{l}\frac{1}{\sqrt{b_{l}}}\phi \left(\frac{x_{i}-a_{k}}{\sqrt{b_{l}}}\right)\frac{\nu }{b_{l}}\chi_{2,\nu }\left(\frac{\nu s_{i}^{2}}{b_{l}}\right). \end{array}$$

Proportionality is resolved by summation over the grid *k*, and we get:

$$\begin{array}{c} {\text{lfdr}}_{i}=P(\theta_{i}=a_{0}|x_{i},s_{i}^{2}),\\ {\text{lfsr}}_{i}=\min\left\{\sum_{k\leq 0}P(\theta_{i}=a_{k}|x_{i},s_{i}^{2}),\sum_{k\geq 0}P(\theta_{i}=a_{k}|x_{i},s_{i}^{2})\right\}. \end{array}$$

It may be helpful to recognize that in contrast to (3), ASH-normal would entail

(4) $$P(\theta_{i}=a_{k}|x_{i},s_{i}^{2})\propto {\hat{g}}_{k}\frac{1}{s_{i}}\phi \left(\frac{x_{i}-a_{k}}{s_{i}}\right),$$

and ASH-*t* would replace the normal density ϕ in (4) with a Student *t* density; in both cases, the ASH-estimated mixing density $\hat{g}$ would come not from (2) but from an objective in which mixing over variances is not explicitly accommodated. The initial implementation of MixTwice invokes unimodality shape constraint, but not symmetry, and, for computational convenience, allows that a random subset of the testing units is used in the optimization. We investigate this approximation in Supplementary Material.

## 3 Simulation study

**Fig. 1.**
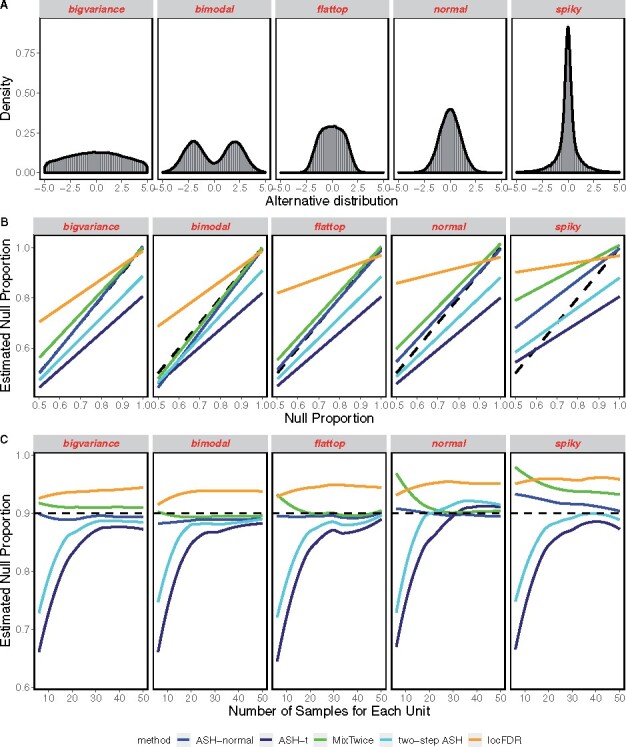
Errors in estimation of *π*_0_. (**A**) Distributions used for $g_{\text{alt}}(\theta )$. (**B**) The estimation of null proportion *π*_0_ in case of equal samples in each group of 10. Methods are distinguished by color, where we report average parameter estimates from 500 simulated datasets. The identity line (dashed) indicates no bias. ASH-normal is an oracle case in which $\sigma_{i}^{2}=1$ is provided to the algorithm. (**C**) Error estimation as the number of observations grows, in $\pi_{0}=0.9$

**Fig. 2.**
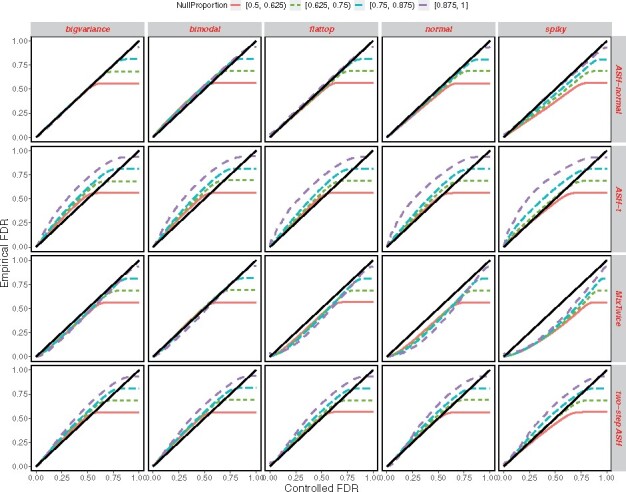
Synthetic data and FDR control: FDRs are shown by different methods (rows) under different alternative distributions $g_{\text{alt}}(\theta )$ (columns). Empirical FDR (vertical) is the achieved error rate in the simulation; controlled FDR (horizontal) is the rate targeted by the methodology. Results with different *π*_0_ are coded using different colors. A method tends to inflate FDR over the target level if its curve is greater than the identity line; it is conservative when its curve is dominated by the identity line

We are interested in the performance of MixTwice in scenarios reflecting what might be expected to occur in practice and have performed numerical experiments involving different generative distributions of both effects (*g*) and variances (*h*). Noting the special role of the null value, *θ* = 0, our experiments involve mixtures $g(\theta )=\pi_{0}\delta_{0}+(1-\pi_{0})g_{\text{alt}}(\theta )$, where $\pi_{0}=P(\theta_{i}=0)$ and $g_{\text{alt}}$ provides various ways to distribute mass away from zero. Following Stephens (2017) and Lu and Stephens (2019), we entertain different general shapes, including the so-called *big-variance*, *bi-modal*, *flattop*, *normal* and *spiky*. MixTwice accounts for explicit differences between sample and underlying standard errors, and mixes nonparametrically over these underlying standard errors. Our numerical experiments consider the simplest case in which the data generating *h* is a point mass, a case involving a finite mixture of two values, and also a continuous case of inverse-Gamma-distributed parameters. Patterns in the error of estimation and the hypothesis testing error rates are very comparable across different choices of *h*, and so for simplicity here we report only experiments when this true *h* is a point mass distribution. Figures 1 and 2 summarize, respectively, properties of estimation accuracy and testing error rates. Experiments are based on Gaussian samples with unit variance, *m *=* *1000 peptides, and various sample size settings for the two-group comparison.

If a method tends to overestimate *π*_0_, then power may be reduced; in case of underestimation the FDR may be inflated. Figure 1B focuses on the estimation of this marginal null frequency for one choice of sample size, namely *n *=* *10 observations per group. In each setting of *g* (column), 500 datasets are generated, each drawn after its own *π*_0_ value was uniformly drawn in [0.5,1]. All methods respond appropriately to changes in *π*_0_, though they exhibit different biases; MixTwice tracks the identity line (no bias) case closely in all scenarios except the challenging *spiky* case of $g_{\text{alt}}$. In contrast, locFDR is conservatively biased, tending to over-estimate *π*_0_ in most cases. Our experiments include an oracle case, namely ASH-normal, which takes the underlying standard errors as known. This numerical control helps us gage the magnitude of statistical errors induced by estimation error of the variance profile.

Figure 1C amplifies one case from the second row, when $\pi_{0}=0.9$, and shows how estimation error drops as the sample size per peptide grows. Most methods display a level of convergence in this setting, with MixTwice performing relatively well especially at low sample sizes. Going beyond the estimation of *π*_0_, we compared methods by their 1-Wassertstein error in estimating the entire mixture distribution *g*; MixTwice showed relatively small error in this setting also (data not shown). MixTwice shares with other nonparametric mixture methods the identifiability problem that only an upper bound on *π*_0_ may be reliably estimated from limited data (Efron *et al.*, 2001; Stephens, 2017). This may be appreciated by considering a single unit, *i*, on which the estimated effect ${\hat{\theta }}_{i}$ is a normal deviation from *θ_i_*, say with known variance $\sigma^{2}=1$, and ignoring the second level of mixing. If $g_{\text{alt}}$ concentrates enough mass near *θ *= 0, then the null predictive density ϕ(x), of ${\hat{\theta }}_{i}$, may be partially absorbed by the alternative predictive density: i.e. there may be a *c *>* *0 such that for all *x*, $c\phi (x)\leq \int \phi (x-\theta )g_{\text{alt}}(\theta )d\theta$, in which case an amount $c(1-\pi_{0})$ of putatively alternative mass could be swapped into the null component without changing the marginal predictive density. Sampling scenarios that allow for decreasing standard errors for at least a fraction of the units resolve this methodological issue. We can show, for symmetric $g_{\text{alt}}$ for example, that the gap *c* vanishes to zero as the standard error *σ* similarly converges (see Supplementary Material). This is consistent with numerical behavior of MixTwice in large samples (Fig. 1C), and is also consistent with work on mixture identification as information per unit increases (Aragam *et al.*, 2020; Ritchie *et al.*, 2020).

Figure 2 confirms that most methods are controlling FDR as advertised. The empirical FDR is plotted against the controlled rate; the latter is the nominal target FDR value where we threshold the lfdr’s; the former is what is evident from knowing the simulation states (in other terminology, it is the average, over simulated datasets, of the false discovery proportion). Colored lines are used to distinguish different levels of *π*_0_, when the signal is dense (with a lower null proportion *π*_0_) or when the signal is sparse (with a higher null proportion *π*_0_). Recall we simulated independent datasets each governed by a randomly chosen *π*_0_ from [0.5,1]. In order to visualize the results, we stratified datasets into four groups and averaged internally: $0.5\leq \pi_{0}\leq 0.625,\;0.625\leq \pi_{0}\leq 0.75,\;0.75\leq \pi_{0}\leq 0.875,\;0.875\leq \pi_{0}\leq 1$. The FDR inflation by ASH-t at high *π*_0_ is evident in this simulation.

## 4 Empirical studies

### 4.1 Antibodies in RA

RA is a chronic autoimmune disease characterized by inflammation and pain, primarily in the joints. RA patients produce autoantibodies against many different ‘self’ proteins. Most famously, they generate antibodies against proteins in which arginine amino acids have been post-translationally modified to citrullines (Schellekens *et al.*, 1998), as well as antibodies that bind to antibodies, called RF (Waaler, 2009). Both autoantibody types appear to be pathogenic (Sokolove *et al.*, 2014) and both are used diagnostically (Aletaha *et al.*, 2010), the former detected by the anti-CCP test. Most RA patients make both autoantibody types (CCP^+^ RF^+^ RA), but some have only one type like in CCP^+^ RF− RA. Little is known about why CCP^+^ RF^+^ versus CCP^+^ RF− RA develops. However, a better understanding of the autoantibody repertoires in each RA subset could provide insights, a task for which peptide arrays are perfect.

The custom high-density peptide array reported in Zheng *et al.* (2020) probed 172 828 distinct 12 amino acid length peptides derived from 122 human proteins suspected to be involved in RA, including peptides in which all arginines were replaced by citrullines. We reconsider here two distinct comparisons from that study, namely the comparison between CCP^+^ RF− RA patients and controls, and a second comparison between CCP^+^ RF^+^ RA patients and controls, in which differential signals are much stronger. Both comparisons have 12 subjects in each group. To assess reproducibility, we take advantage of a second peptide array dataset derived from an independent set of eight controls and eight CCP^+^ RF^+^ RA patients.

### 4.2 CCP^+^RF− RA: weak signals

**Fig. 3.**
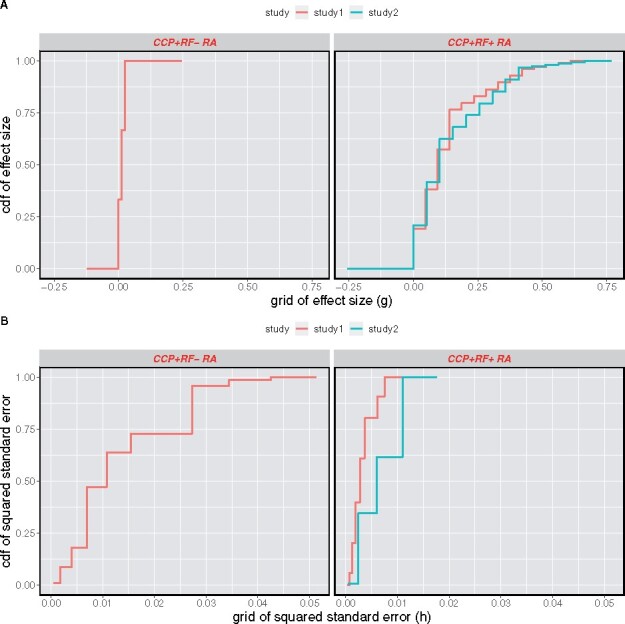
Estimated mixing distributions: for both effect distribution *g* (**A**) and squared-standard-error distribution *h* (**B**), shown are the maximum likelihood estimated mixing distributions as cumulative distribution functions (cdfs) in double natural log scale. The CCP^+^ RF− RA example is shown on the left and the two CCP^+^ RF^+^ RA examples are on the right

We applied MixTwice to fit the shape-constrained mixture model of Section 2. Fitted mixing distributions are visualized in Figure 3 and provide a measure of the magnitude of changes in mean antibody levels as well as the magnitude of sampling variation. For example, the effect-size distribution estimates no probability for effects larger than 0.037. Also, the median standard error is 0.10 (squared standard error 0.01), which is large compared to the probable effect sizes.

**Fig. 4.**
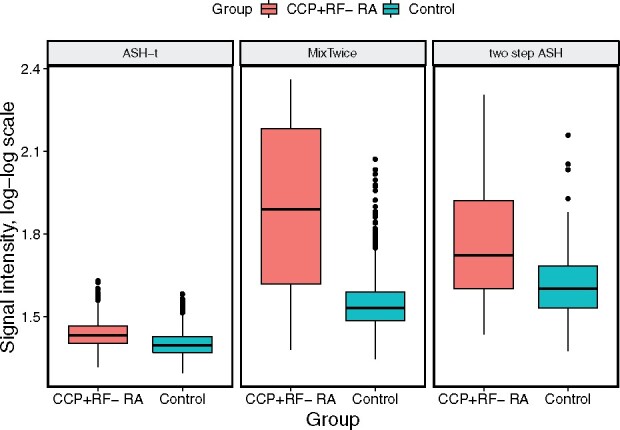
Signal intensity of differentially abundant peptides: boxplots show averaged signal values on double natural log scale (both CCP^+^ RF− RA and control subjects) for peptides found by ASH-t (76 peptides), MixTwice (44 peptides) and two-step ASH (11 peptides) all discovered at 10% FDR

In Section 1, we presented summary counts of peptides identified at 10% FDR that exhibits differential binding between CCP^+^ RF− RA patients and non-RA controls. MixTwice, ASH-t and two-step ASH distinguish themselves in being the only methods among many standard large-scale tools to populate nonempty lists of discovered peptides at that FDR level. Recognizing that the magnitude of signal intensities on the peptide array is an important aspect of downstream analysis, Figure 4 shows a summary of the identified peptides by various methods. Notably, MixTwice and two-step ASH detect peptides in this case with higher average signal intensity than ASH-t; these may correspond to higher antibody abundance or affinity and potentially easier validation. ASH-t tends to select peptides with low standard errors, even when the estimated effects are very low.

**Fig. 5.**
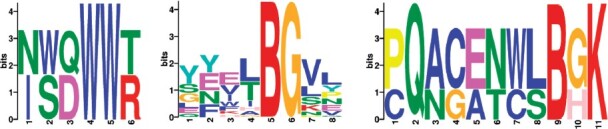
Motif logo for significant peptides in CCP^+^ RF− RA: consensus sequences were generated using online software MEME Suite (Bailey *et al.*, 2009) and the significant peptides from the different methods: ASH-t (left), MixTwice (middle) and two-step ASH (right). Each position of the motif logo represents the empirical distribution of amino acids at that site, with size proportional to frequency. *B* found in the middle and right panels is citrulline, a post-transitionally modified arginine. The overall height of each stack is an information measure (bits) related to the concentration of the empirical distribution on its support

Interestingly, the 44 peptides found by MixTwice have a strong pattern in their peptide sequences: all are citrulline (*B*)-containing peptides (which would be predicted for CCP^+^ RA patients) and contain citrulline next to glycine (*B*-*G* or *G*-*B*), as shown in the motif in Figure 5. Binding of antigens in which citrulline is next to glycine is consistent with a growing body of literature on the reactivity of anti-citrullinated protein antibodies in RA (e.g. Burkhardt *et al.*, 2002; Steen *et al.*, 2019; Szarka et al., 2018; Zheng *et al.*, 2020).

As a further negative control calculation, we applied MixTwice to each of 500 permuted datasets obtained by fixing the peptide data and randomly shuffling the 24 subject labels (12 control, 12 CCP^+^ RF− RA). In 493 cases, the 10% FDR list is empty; 6 cases find a single peptide and 1 case finds 2 peptides at this threshold.

Among a number of large-scale testing methods applied to the CCP^+^ RF− RA example, MixTwice identifies comparatively a large number of statistically significant peptides. In contrast to other methods, these peptides contain patterns in their amino acid sequences consistent with emerging evidence on this disease, and they correspond to relatively high fluorescence intensity measurements. Together, these observations provide some assurance that the MixTwice findings are not artifacts.

### 4.3 CCP^+^RF^+^RA: strong signals

One of the findings from Zheng *et al.* (2020) concerns the extensive antibody-profile differences between RA patients who are positive for both biomarkers (CCP^+^ RF^+^) and control subjects. Statistically, it represents an interesting nonsparse, large-scale testing situation, and the immunological mechanisms driving this remain only partially understood. To check the reproducibility of peptide array findings, a new experiment was performed using the same procedures and 172 828 peptide array to detect IgG binding as in Zheng *et al.* (2020), but with serum samples from 16 different subjects: 8 CCP^+^ RF^+^ RA and 8 controls. CCP^+^ RF^+^ RA and control subjects were similar in regards to age, sex, race, ethnicity and overall health. Preprocessing followed the same protocol and provided a dataset (*study 2*) for us to look at reproducibility of large-scale hypothesis testing methods.

**Fig. 6.**
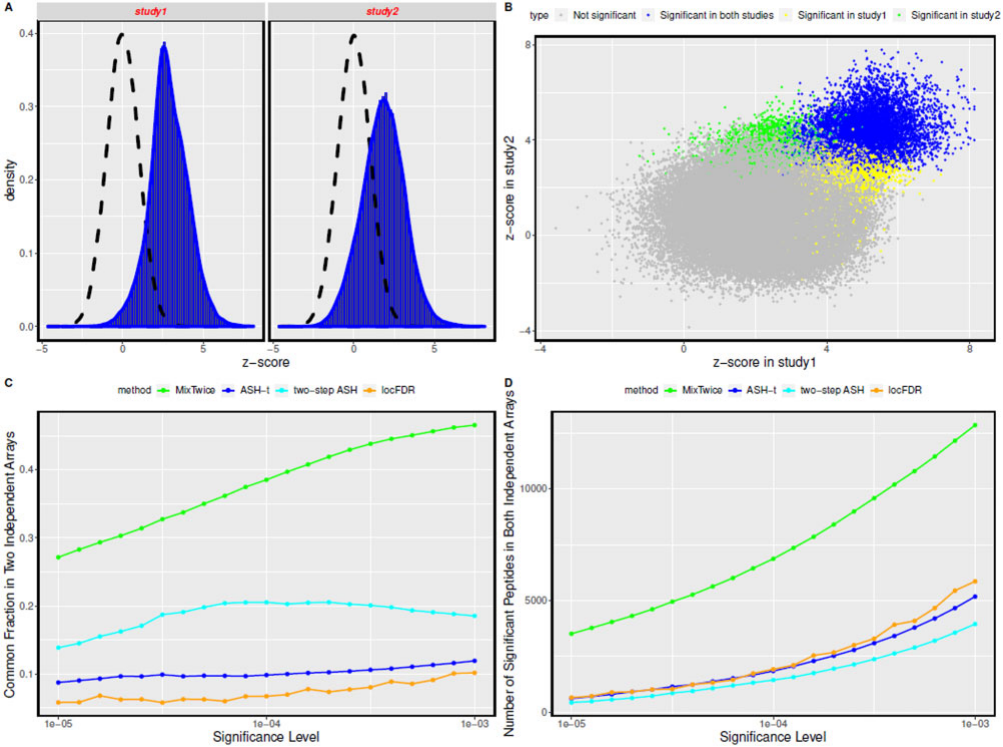
Reproducibility comparison. (**A**) Empirical z-score distributions for CCP+RF+RA versus control at 172 828 peptides in two independent studies. The scatterplot in (**B**) highlights peptides identified uniquely at 0.1% FDR by MixTwice in either study (yellow, green) and those reproducibly found in both studies (blue). Metrics in (**C**) and (**D**) compare performance of MixTwice as a function of FDR threshold

Z-score histograms in Figure 6A show that both studies reveal extensive increased antibody binding in the CCP^+^ RF^+^ RA group. The scatterplot in Panel B reveals concordance between the studies on this *z*-score metric. The color-coding highlights discovered peptides at the 0.1% FDR method by MixTwice, both uniquely in one study (green or yellow) and reproducibly in both studies (blue). Of course, MixTwice uses more information than is in the *z*-score summary, but the scatterplot provides a convenient visualization. The lower panels in Figure 6 compare reproducibility statistics of different testing methods at various FDR thresholds. Denoting by $\ell_{j}(\alpha )$ the list of significant peptides in study *j* and FDR level *α*, we have $\left|\ell_{1}(\alpha )\cap \ell_{2}(\alpha ))\right|$ as the number of peptides identified in both studies (Panel D) and $\frac{\left|\ell_{1}(\alpha )\cap \ell_{2}(\alpha ))\right|}{\left|\ell_{1}(\alpha )\cup \ell_{2}(\alpha ))\right|}$ as the common fraction (Panel C). By connecting separate, independent studies of the same group difference, these statistics measure the reproducibility of various large-scale testing methods. MixTwice shows substantially better reproducibility than other testing methods, such as ASH-t, two-step ASH and locFDR in this example.

## 5 Discussion

High-throughput biomedical experiments, such as those involving peptide arrays and immunological studies, continue to provide challenging problems for large-scale hypothesis testing. Readily applied techniques, such as *q*-value, locFDR, IHW and ASH are often very effective at reporting lists of testing units (peptides) showing statistically significant effects at a targeted FDR. In the case of high-density peptide arrays, we find several examples where these tools are deficient. One issue is the number of testing units, which is an order of magnitude larger than what is seen in transcript studies, for example. In the CCP^+^ RF− RA comparison, most existing tools exhibit low power, which may stem in part from when they intervene in the data analysis. Methods that intervene earlier have access to more information and thereby may gain some advantage. The risk to intervening early is that more assumptions may be required to deliver relevant testing statistics (e.g. lfdr, lsdr). We rely on external validation, such as on sequence properties of the identified peptides, to assess practical utility. The CCP^+^ RF^+^ RA example showcases a situation where power is high by all methods, and the differences boil down to how testing units are prioritized. The proposed MixTwice procedure shows impressive reproducibility in this case.

Structurally, MixTwice is similar to the ASH method for large-scale testing: it aims to estimate a mixing distribution of effects in an empirical Bayesian formulation. It adopts ASH’s non-parametric, shape-constrained model for effects, but deviates from that approach by incorporating a second mixing layer over underlying effect-variance parameters. A number of methodological issues deserve further study. For example, MixTwice treats the sampling model of squared standard errors as chi-square on a design-based degrees of freedom, which is rooted in a normal-data model. We expect that suitable transformation of the original data will make this treatment reasonable; for example, Zheng *et al.* (2020) proposed a double-log transform to stabilize variance. An interesting alternative is to use a bootstrap scheme to assess the sampling distributions directly, in order to thereby estimate the degrees of freedom that would be justified asymptotically for non-normal cases.

There are computational issues that warrant further investigation. The objective function (2) may not be convex in the pair of arguments (*g*, *h*). Numerical experiments indicate good performance of the augmented Lagrangian optimization approach in a range of scenarios, though alternative approaches may have benefits. For example, the conditional optimizations of *g* given *h* or *h* given *g* are both convex, though attempts so far to leverage this have been less computationally efficient than the augmented Lagrangian method. Related to this are questions of grid sizes *K* and *L*, which have to balance fidelity to the data and computational efficiency.

Though our presentation has focused on the classical two-group comparison problem, it should be evident that the core methodology is not restricted to this case. Estimated effects *x_i_*, for example, could arise from a contrast of interest after adjusting for blocking variables or other covariates. These will be useful to consider as we expect them to emerge in experiments that further investigate mechanisms of immune-system disregulation.

Finally, we point out that other forms of information may be usefully integrated with the testing methodology. Peptides tile proteins, though we have treated them as anonymous testing units. More sophisticated peptide prioritization could leverage amino-acid structure, protein content or other features of the immunological context.

## Funding

This work was supported by the Peer Reviewed Medical Research Program (US Army Medical Research, W81XWH1810717) as well as by the University of Wisconsin-Madison, Office of the Vice Chancellor for Research and Graduate Education with funding from the Wisconsin Alumni Research Foundation to M.A.S. and also National Institutes of Health [R01 GM102756], National Institutes of Health [P50 DE026787] and NSF [1740707] supporting M.A.N. I.M.O. acknowledges support by the Clinical and Translational Science Award (CTSA) program, through the National Institutes of Health National Center for Advancing Translational Sciences (NCATS) [UL1TR002373 and KL2TR002374]. This research was also supported by the Data Science Initiative grant from the University of Wisconsin-Madison Office of the Chancellor and the Vice Chancellor for Research and Graduate Education (with funding from the Wisconsin Alumni Research Foundation) (I.M.O.). The authors acknowledge Sean McIlwain for assistance with reproducibility calculations, and thank a referee and Associate editor for extremely useful comments on an earlier draft.

*Conflict of Interest:* none declared. 

## Data availability

All data used in this study are available at github.com/wiscstatman/MixTwice/data/

## Supplementary Material

btab162_Supplementary_Data
