# Supplementary material for: MixTwice: large-scale hypothesis testing for peptide arrays by variance mixing
Source: Bioinformatics. 2021 Mar 8;37(17):2637–43. doi: 10.1093/bioinformatics/btab162 (PMC8428605; doi:10.1093/bioinformatics/btab162)
Supplement: btab162_Supplementary_Data [file btab162_supplementary_data.pdf]

Supplementary Material

Main document: *MixTwice: Large-scale hypothesis testing for peptide arrays by variance mixing*

Authors: Zheng, Mergaert, Ong, Shelef, and Newton

Version: February 14, 2021

1 Gradient and Hessian of optimization objective

We derive the gradient and Hessian of the log-likelihood equation, $l(g,h)$. Recall the definition of $l(g,h)$:

$$
\begin{aligned}
l(g,h) &= \sum_{i=1}^{m} \log p(x_i, s_i^2|g,h) \\
p(x_i, s_i^2|g,h) &= \sum_k \sum_l g_k h_l \frac{1}{\sqrt{b_l}} \phi\left(\frac{x_i - a_k}{\sqrt{b_l}}\right) \frac{\nu}{b_l} \chi_{2,\nu}\left(\frac{\nu s_i^2}{b_l}\right).
\end{aligned}
$$

To simplify notation we use $c_{i,k,l}$ to denote the prior, mixture density of sample i on the grid a_k, b_l, and we let d_i denote the observation density:

$$
\begin{aligned}
c_{i,k,l} &:= \frac{1}{\sqrt{b_l}} \phi\left(\frac{x_i - a_k}{\sqrt{b_l}}\right) \frac{\nu}{b_l} \chi_{2,\nu}\left(\frac{\nu s_i^2}{b_l}\right) \\
d_i &:= p(x_i, s_i^2|g,h) = \sum_k \sum_l g_k h_l \; c_{i,k,l}.
\end{aligned}
$$

Consider the parameter vector (g,h) of length $(2K+1)+L$, where the first $2K+1$ components are for the effect mixing probabilities, $g = (g_k)$, and

the remaining L components are for variance mixing probabilities $h = (h_l)$. The quantities $c_{i,k,l}$ depend on the data, the support points but not the probabilities g and h.

Constraints are critical to the optimization; of course all elements of g and h must be positive and sum to unity. We also impose a unimodality constraint on g. But in deploying the augmented Lagrangian method, these constraints act on the differentiable function $l(g, h)$, which we consider initially as varying freely over $2K + L + 1$ Euclidean space. The gradient of $l(g, h)$ is a column vector of length $(2K + 1) + L$ with the following format:

$$\nabla l(g,h) \;=\; \left(\left(\frac{\partial l(g,h)}{\partial g}\right)', \left(\frac{\partial l(g,h)}{\partial h}\right)'\right)'$$

where each component has the explicit form:

$$\frac{\partial l(g,h)}{\partial g_k} \;=\; \sum_{i=1}^{m} \frac{1}{d_i} \sum_{l} h_l \; c_{i,k,l}$$
$$\frac{\partial l(g,h)}{\partial h_l} \;=\; \sum_{i=1}^{m} \frac{1}{d_i} \sum_{k} g_k \; c_{i,k,l}.$$

The Hessian of $l(g, h)$ is a $(2K + 1) + L$ by $(2K + 1) + L$ matrix:

$$\nabla^2 l(g,h) = \begin{pmatrix} A & B \\ B' & C \end{pmatrix}$$

where matrix A ($2K + 1$ by $2K + 1$) contains second derivative with respect to g, matrix C (L by L) contains second derivative with respect to h and

matrix B ($2K+1$ by L) contains second derivative with respect to g and h.

For entries of matrix A:

$$\frac{\partial^2 l(g,h)}{\partial g_k^2} = -\sum_{i=1}^{m} \frac{1}{d_i^2} \left(\sum_l h_l \; c_{i,k,l} \right)^2$$
$$\frac{\partial^2 l(g,h)}{\partial g_{k_1} \partial g_{k_2}} = -\sum_{i=1}^{m} \frac{1}{d_i^2} \left(\sum_l h_l \; c_{i,k_1,l} \right) \left(\sum_l h_l \; c_{i,k_2,l} \right).$$

For entries of matrix C:

$$\frac{\partial^2 l(g,h)}{\partial h_l^2} = -\sum_{i=1}^{m} \frac{1}{d_i^2} \left(\sum_k g_k \; c_{i,k,l} \right)^2$$
$$\frac{\partial^2 l(g,h)}{\partial h_{l_1} \partial h_{l_2}} = -\sum_{i=1}^{m} \frac{1}{d_i^2} \left(\sum_k g_k \; c_{i,k,l_1} \right) \left(\sum_k g_k \; c_{k,l_2} \right).$$

For entries of matrix B:

$$\frac{\partial^2 l(g,h)}{\partial g_k \partial h_l} = \sum_{i=1}^{m} \frac{1}{d_i^2} \left(c_{i,k,l} \; d_i - \sum_l h_l \; c_{i,k,l} \sum_k g_k \; c_{i,k,l} \right).$$

2 Random subsampling

The optimization to compute $\hat{g}$ and $\hat{h}$ becomes computationally challenging as the number of testing units increases. `MixTwice` provides an option for users to use a randomly-selected subset of testing units to obtain the fitted distributions. Here we illustrate the compute-time improvements associated with relatively little degradation in the quality of the estimates.

We use the CCP+RF+ RA example to illustrate the random subsampling properties in terms of estimation error and computational benefit. Relative to the estimate obtained from half the units, we evaluate the discrepancy in

distribution estimation and the user's CPU time (with Inter(R) Core(TM) i5-7400HQ CPU processor) when the `prop`, the proportion of testing units used to fit the distribution, changes. We use 1-Wasserstein distance between two cumulative distribution functions as the metric to evaluate the discrepancy from the case when `prop` $= 0.5$ as benchmark.

Figure S1 summaries the result. Panel A highlights the estimation of $\hat{g}, \hat{h}$ when `prop` $= 0.5, 0.1, 0.01$ where the estimations are quite similar. Panel B shows how the discrepancy decreases when the proportion of testing units used to fit the distribution increases. Note that even when `prop` $= 0.01$, the discrepancy is quite small (error in $\hat{g}$ less than 0.02 and error in $\hat{h}$ only 10^-4). Panel C shows the computational benefits.

3 On identifiability

On units i with a fixed, known standard error σ, the mixing model for effects θ_i is puts point mass at 0, with probability π_0, and distributes the remaining mass according to some distribution g_{alt}, which in the following is treated as a density function with respect to Lebesgue measure. Ignoring mixing over σ (according to h), the predictive density of estimator $\hat{\theta}_i$, at argument x, is

$$\pi_0 \frac{1}{\sigma}\phi\left(\frac{x}{\sigma}\right) + (1-\pi_0)\int \frac{1}{\sigma}\phi\left(\frac{x-\theta}{\sigma}\right) g_{\text{alt}}(\theta)\, d\theta$$

where ϕ is the standard normal density reflecting Gaussian errors of the estimators. The alternative effect density g_{alt} need not be zero in neighborhoods of the null, in which case it may happen that there exists a *gap* $c = c_\sigma > 0$ for which, for all x,

$$c_\sigma \frac{1}{\sigma}\phi\left(\frac{x}{\sigma}\right) \leq \int \frac{1}{\sigma}\phi\left(\frac{x-\theta}{\sigma}\right) g_{\text{alt}}(\theta)\, d\theta. \tag{1}$$

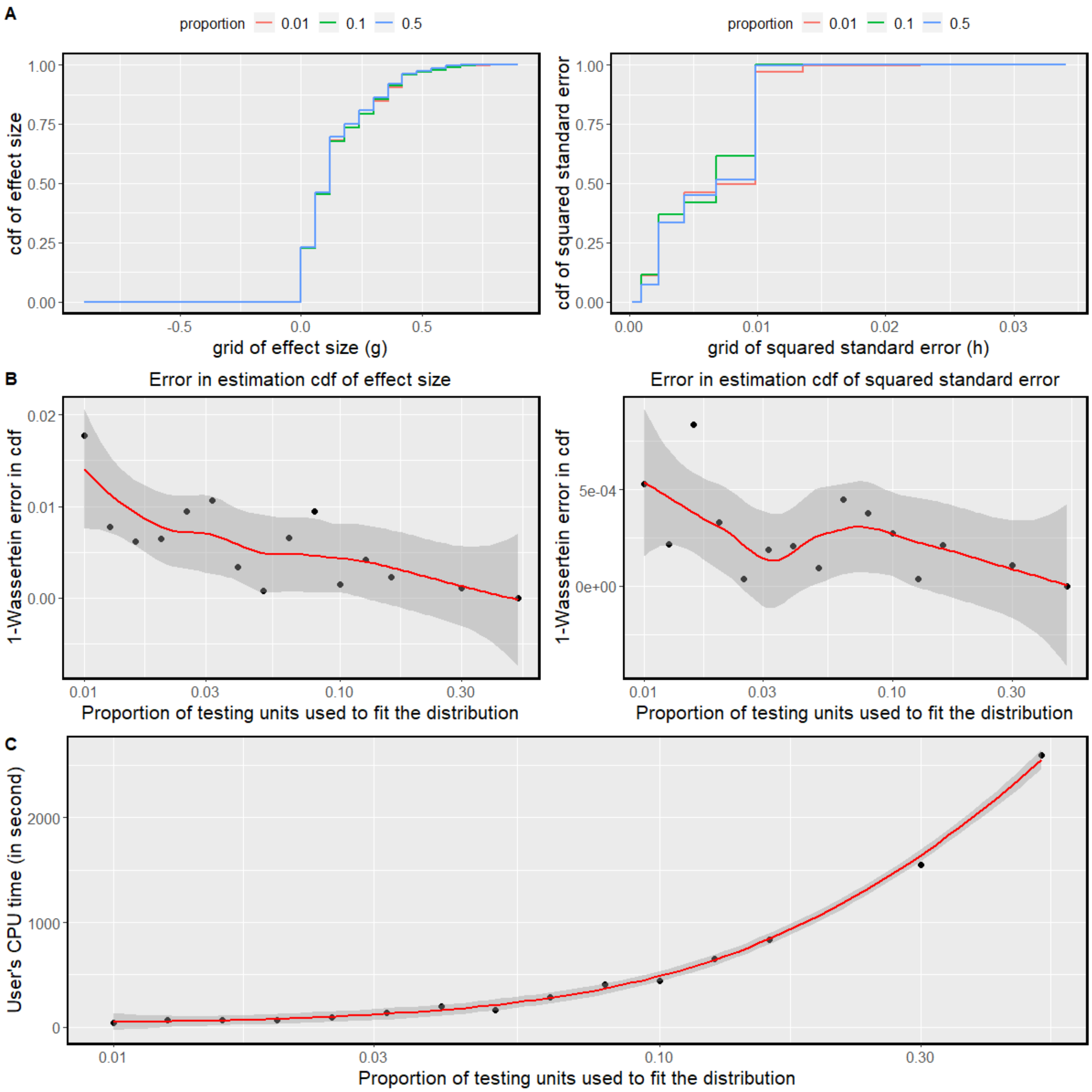

Supplementary Figure S1: **How does random subsampling influence estimation accuracy and computational efficiency?** Panel A shows the estimation in $\hat{g}, \hat{h}$ when various proportions of the units are used for estimation. Panel B shows the 1-Wasserstein discrepancy (between estimate at that proportion and estimate from half the units) as a function of subsampling proportion. Panel C shows the corresponding CPU time.

If there is a gap, the alternative predictive density contains within it a shrunken version of the null. The problem with such a gap is well known; an amount $c_\sigma(1-\pi_0)$ of mass from the alternative predictive component may be pushed into the null component, with no effect on the marginal predictive density. A small gap emerges in cases such as *spiky* (Figure 1, main) where g_{alt} concentrates substantial mass near the null value. This constitutes an identifiability issue, however, we find that the gap is small or nonexistent in many cases, and anyway can be shown to converge to zero when σ converges to zero. To see this feature, rearrange (1) to see that for all x we require

$$c_\sigma \leq \int \exp\left\{\frac{1}{2\sigma^2}(2\theta x - \theta^2)\right\} g_{\text{alt}}(\theta)\, d\theta$$

The bound on the right depends on x; differentiating in x, under the integral gives

$$\int \frac{\theta}{\sigma^2} \exp\left\{\frac{1}{2\sigma^2}(2\theta x - \theta^2)\right\} g_{\text{alt}}(\theta)\, d\theta.$$

Notice that if g_{alt} is symmetric, then at $x = 0$ this derivative is zero, and so

$$c_\sigma \leq \int \exp\left\{\frac{-\theta^2}{2\sigma^2}\right\} g_{\text{alt}}(\theta)\, d\theta.$$

Taking appropriate limits in σ towards 0 shows that c_σ must vanish, which will happen with increasing amounts of information per unit. The question of mixing over σ using the second mixing distribution h is not directly addressed by the above computations. However, we would predict from them that as the estimated mixing distribution $\hat{h}$ concentrates more of its mass on small standard errors, then inferences about effects θ_i will be ever more reliable.
